# Cycling to Meet Fate: Connecting Pluripotency to the Cell Cycle

**DOI:** 10.3389/fcell.2018.00057

**Published:** 2018-06-19

**Authors:** Lamuk Zaveri, Jyotsna Dhawan

**Affiliations:** ^1^Institute for Stem Cell Biology and Regenerative Medicine, Bangalore, India; ^2^CSIR - Centre for Cellular and Molecular Biology, Hyderabad, India; ^3^Manipal Academy of Higher Education, Manipal, India

**Keywords:** cell cycle, pluripotency, embryonic stem cells, reprogramming, induced pluripotent stem cells

## Abstract

Pluripotent stem cells are characterized by their high proliferative rates, their ability to self-renew and their potential to differentiate to all the three germ layers. This rapid proliferation is brought about by a highly modified cell cycle that allows the cells to quickly shuttle from DNA synthesis to cell division, by reducing the time spent in the intervening gap phases. Many key regulators that define the somatic cell cycle are either absent or exhibit altered behavior, allowing the pluripotent cell to bypass cell cycle checkpoints typical of somatic cells. Experimental analysis of this modified stem cell cycle has been challenging due to the strong link between rapid proliferation and pluripotency, since perturbations to the cell cycle or pluripotency factors result in differentiation. Despite these hurdles, our understanding of this unique cell cycle has greatly improved over the past decade, in part because of the availability of new technologies that permit the analysis of single cells in heterogeneous populations. This review aims to highlight some of the recent discoveries in this area with a special emphasis on different states of pluripotency. We also discuss the highly interlinked network that connects pluripotency factors and key cell cycle genes and review evidence for how this interdependency may promote the rapid cell cycle. This issue gains translational importance since disruptions in stem cell proliferation and differentiation can impact disorders at opposite ends of a spectrum, from cancer to degenerative disease.

## Introduction

Embryonic stem cells (ES) are derived from the inner cell mass of the blastocyst and can be cultured indefinitely *in vitro* while still remaining pluripotent (Evans and Kaufman, 1981; Thomson et al., 1998). They can give rise to the three germ layers Endoderm, Mesoderm and Ectoderm *in vivo* when transplanted into mice or *in vitro* under appropriate culture conditions. The ability to give rise to a range of different cell types has made ES cells highly attractive for their potential use in regenerative medicine both as a potential source of differentiated cells for replacement therapies, but more immediately as an excellent model for understanding developmental programming as a means to eventually targeting endogenous adult stem cells *in vivo*.

ES cells express a set of genes characteristic of the pluripotent state including transcription factors such as Oct-3/4, Sox2, and Nanog (Nichols et al., 1998; Niwa et al., 2000; Avilion et al., 2003; Mitsui et al., 2003; Chambers et al., 2007). These transcription factors are essential in maintaining the pluripotent state and when expressed in other cell types, can also confer enhanced stemness as seen in cancer stem cells (Liu A. et al., 2013; Jeter et al., 2015; Wang and Herlyn, 2015). By expressing a combination of four transcription factors that are also expressed in ES cells namely, Oct-3/4, Klf4, Sox2 and c-Myc (Yamanaka factors), it is possible to “reprogram” somatic cells to a pluripotent state (Takahashi and Yamanaka, 2006; Takahashi et al., 2007).

ES cells can divide rapidly, with doubling times ranging from 8 to 10 h (Solter et al., 1971; Power and Tam, 1993) as compared to somatic cells such as embryonic fibroblasts with doubling times of ~20 h or more (Savatier et al., 1994; Stead et al., 2002; Becker et al., 2006). The rapid proliferation in ES cells is brought about by a massive rearrangement in regulators of the cell cycle, including modifications to checkpoints, altered expression patterns of key cell cycle genes and altered metabolic regulation (Shyh-Chang et al., 2013). Coupled with the limitless propensity to self-renew and repress genes that control lineage commitment and differentiation, the unique ES cell cycle is at the heart of stem cell function.

Due to this highly interlinked network, dissecting the mechanisms regulating the ES cell cycle is challenging. By using a combination of cell synchronization, directed differentiation, reprogramming to a pluripotent state, newer imaging techniques, improved resolution of a variety of methods targeted at single cells and better automation algorithms, we now have a more detailed understanding of the many unique regulatory aspects controlling the ES cell cycle. Several recent reviews have highlighted the connections between developmental programming and proliferative control (Kareta et al., 2015b; Soufi and Dalton, 2016). In this review, we discuss the recent discoveries which highlight how deviation of the ES cell cycle from the somatic cell cycle allows both rapid proliferation and retention of stemness. We also discuss how the cell cycle changes along with the pluripotent state, during the course of embryonic development. Finally, we examine the various links between transcription factors that maintain pluripotency in ES cells and those that regulate the rapid cell cycle.

## The truncated mES cell cycle

The cell cycle is characterized by a complex interplay of Cyclins, Cyclin-dependent kinases (Cdk), Cyclin-dependent kinase inhibitors (Cdkn), pocket proteins of the retinoblastoma family and many accessory factors. This intricate network provides an organized system by which a cell can grow and divide into two daughter cells (Morgan, 1995; Hindley and Philpott, 2013). Depending on the cell type, the time taken by a proliferating cell to divide varies, and is primarily brought about by modulating these regulators of the cell cycle (Harashima et al., 2013).

**Figure 1:**
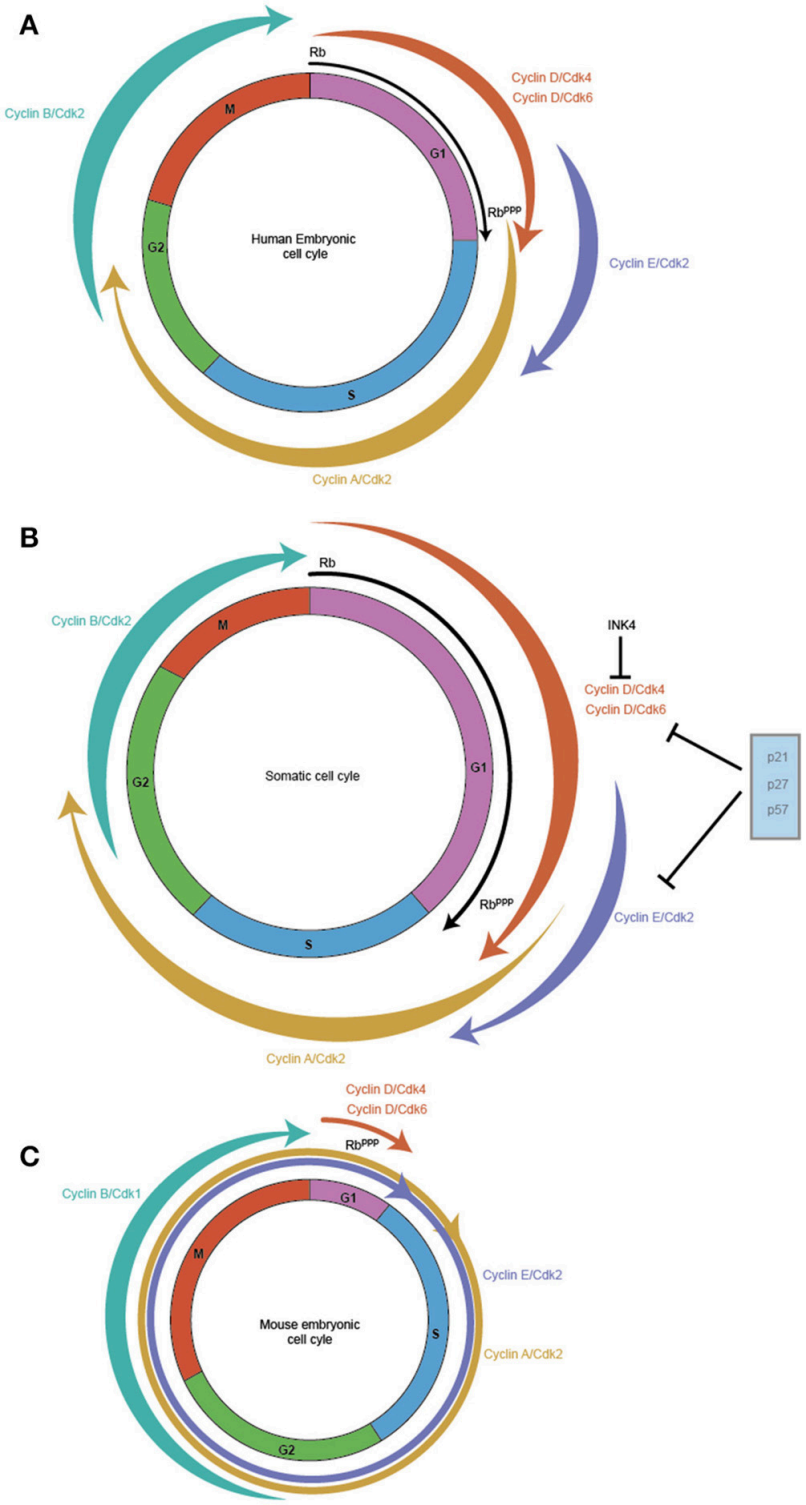
Cell cycles vary between somatic and pluripotent stem cells. Embryonic stem cells exhibit faster proliferation rates, which is reflected in their modified cell cycles. In comparison to the somatic cell cycle in embryonic fibroblasts with a cell cycle duration of ~20 h **(B)** hESC display a cell cycle duration of 15 h **(A)** while in mES, it is shortened to ~10 h **(C)**. The main difference between the three cell cycles is the length of the G1 phase which is highly reduced in mES, with hESC exhibiting a shortened G1 and somatic cells exhibiting a relatively longer G1. The weighted arrows indicate Cyclin-Cdk complex activity, which in somatic cells and hESC exhibit a canonical oscillatory behavior across the cell cycle. In mES, Cyclin B/Cdk1 is the only complex that displays this oscillatory behavior, while Cyclin E/Cdk2, Cyclin A/Cdk2 are active throughout the mES cell cycle and Cyclin D/Cdk4/Cdk6 exhibits very low activity during the reduced G1. RB, the pivotal regulator of the Restriction point in G1 is active (RB) at the start of G1 and gets progressively phosphorylated across G1 leading to its inactivation (RB^ppp^), and allows the cell to cross the G1/S checkpoint. mES have a perpetually inactive RB^ppp^, thereby allowing unfettered transit through G1.

A canonical somatic cell cycle defined by studies in cultured fibroblasts such as NIH 3T3 consists of a DNA synthesis stage (S phase) and the cell division phase (M phase) interspersed by two gap phases called G1 (between M phase and S phase) and G2 (between S phase and M phase) (Hindley and Philpott, 2013). The cell cycle is primarily regulated by the action of Cyclin-Cdk complexes, which exhibit an oscillatory activity that activates and represses crucial regulators of the cell cycle to promote transitions from one phase to the next. Cyclin D along with its Cdk partners Cdk4/6 exhibits high activity in the G1 phase, while Cyclin E with its partner Cdk2 is active during the late G1 phase and S phase. Cyclin A with Cdk2 is predominantly active in the S phase and G2, while Cyclin B with Cdk1 regulates G2 and M phase. The ordered appearance and disappearance of these regulatory proteins is required to ensure that DNA synthesis precedes cell division, enforcing the mechanisms that control precise genome size and integrity. Thus, the oscillatory activity of the related but distinct Cyclin-Cdk complexes is thought to drive the cell cycle unidirectionally, by a ratcheting mechanism involving the activation and destruction of distinct targets that regulate characteristic aspects of each cell cycle phase (Hindley and Philpott, 2013) (Figure 1B).

By contrast, mouse embryonic stem cells (mES) cells exhibit a cell cycle in which the G1 phase is highly reduced allowing the cell to rapidly shuttle between cell division (M phase) and DNA synthesis (S phase) (Mac Auley et al., 1993; Stead et al., 2002; Fujii-Yamamoto et al., 2005; Lange and Calegari, 2010) (Figure 1C). Further, the typical oscillatory activity of Cyclin-Cdk complexes seen in a somatic cell cycle is absent, yet mES cells still cycle (Soufi and Dalton, 2016). During the reduced G1 phase seen in mES, Cyclin D1, and D3 are expressed at low levels with Cdk6 being the predominant Cdk partner, but whether cause or consequence of the reduced G1 phase has been difficult to determine (Faast et al., 2004; Ter Huurne et al., 2017). In contrast, Cyclin E/Cdk2, and Cyclin A/Cdk2 activity is high throughout the ES cell cycle to the point that their activity is considered cell cycle independent (Stead et al., 2002; Fujii-Yamamoto et al., 2005; Ter Huurne et al., 2017). The mitotic cyclin, Cyclin B activity is the only exception, its activity along with Cdk1 peaks during G2/M phase and is low during the other phases of the mES cell cycle (Stead et al., 2002; Fujii-Yamamoto et al., 2005; Ter Huurne et al., 2017).

A key regulator of the G1 phase is the retinoblastoma protein (RB) which controls the Restriction point thereby preventing entry into S phase (Pardee, 1989; Weinberg, 1995). This control is brought about by modifying the phosphorylation status of RB. When a cell enters G1, RB is in an active (unphosphorylated) state and blocks transcription of key G1/S phase genes, preventing passage across the Restriction point (Weinberg, 1995; Lundberg and Weinberg, 1998). Phosphorylation of RB across the G1 phase reduces its inhibitory activity, allowing cells to start a series of reactions which allows it to overcome the Restriction point and finally enter the S phase (Weinberg, 1995; Lundberg and Weinberg, 1998).

RB binds with E2F to control the expression of G1/S phase cell cycle regulators such as Cyclin E, Cyclin A, and Cdk2 (Chen et al., 2009). The E2F family of transcription factors which consists of eight members has been classically divided into two sub categories of “activators” and “repressors” (Chen et al., 2009). During the early phase of G1, active RB (unphosphorylated) forms a repressive complex with repressor E2Fs and binds to the promoters of target genes, recruiting histone deacetylases to repress their transcription (Brehm et al., 1998; Luo et al., 1998). RB also directly suppresses the activity of activator E2Fs by binding and preventing the formation of an activator complex (Helin et al., 1993).

In mES, RB is in a perpetually hyperphosphorylated (inactive) form due to the high activity of Cyclin-Cdk which phosphorylate RB, coupled with a reduction in phosphatases such as protein phosphatase (PP-1) (Stead et al., 2002; Kolupaeva and Janssens, 2013). As RB is inactive, there is no repression of activator E2F and repressor E2Fs do not form a repressive complex with RB (Stead et al., 2002). This leads to high expression levels of Cyclin E/Cyclin A & Cdk2 which in turn further inhibits RB activity (Stead et al., 2002). Loss of RB repressive activity leads to the inactivation of the G1-S checkpoint, allowing mES cells to rapidly enter S phase almost immediately after cell division.

Another layer of regulation of Cyclin-Cdk activity is through the action of Cyclin dependent kinase inhibitors (Cdkns). There are two main classes of Cdkn, the CIP/KIP family, which consists of p21 (Cip1), p27 (Kip1), and p57 (Kip2) and the INK4/Arf (Inhibitors of Cdk4) which consists of p16 (Ink4a), p15 (Ink4b), p18 (Ink4c), and p19 (Ink4d/Arf) (Sherr and Roberts, 1999). The CIP/KIP family has a broader inhibitory activity and can bind to both Cyclins and Cdk. They can regulate the activity of Cyclin D, Cyclin E, and Cyclin A (Sherr and Roberts, 1999). On the other hand, the INK4 family specifically inhibits Cdk4 and Cdk6 activity by interfering with their binding with Cyclin D (Sherr and Roberts, 1999). Importantly, mES cells do not express any Cdkns which contributes to the high activity of Cyclin-Cdk (Stead et al., 2002; Fujii-Yamamoto et al., 2005). Indeed, Cyclin D3/Cdk6 in mES cells are immune to the effects of p16, though the mechanism is still not clearly understood (Faast et al., 2004). The combined effect of high activity of Cyclin E/A/Cdk2, inactive RB and absence of Cdkns establishes the rapid cell cycle typical of mES cells (Savatier et al., 1994; Stead et al., 2002).

As mES cells start to differentiate, the Cyclin-Cdks begin to exhibit oscillatory behavior, the RB checkpoint is enabled and the Cdkns are expressed. These changes combine to confer gradual increase in the length of the G1 and subsequent increase in cell cycle duration (Lange and Calegari, 2010). The order in which the cell cycle regulators begin to show altered expression during this process is still not clear. To increase the length of G1, activation of either the Cdkns or RB is required, as both these inhibitory regulators function to dampen the effect of Cyclin–Cdk activity. Whether expression of RB or Cdkn is activated first, or both are activated together is not known. The recent development of improved methods to sort cells into discrete phases of the cell cycle (Pauklin and Vallier, 2013; Singh et al., 2016), will aid in resolving this issue.

## Dissecting the ES cell cycle: a changing paradigm requiring new technologies

In culture systems, the cell populations under analysis are generally asynchronous which makes it difficult to study any cell cycle related phenomena as they may be masked by out of phase cells. In order to study cyclical processes, cells are generally synchronized using a variety of methods to enrich for populations at a particular stage of the cell cycle and then “released” to normal conditions to permit transition to the next phase. Synchronisation generates cell populations that are homogeneous with respect to their cell cycle phase, permitting an analysis of phase specific events and their regulation. Common methods for generating synchrony include the use of drugs such as Nocodazole (causes cell cycle arrest at G2/M by preventing formation of the mitotic spindle), serum starvation (causes cell cycle arrest at G0/G1 due to removal of mitogens that prevent activity of the Restriction point) or feedback control through disruption of DNA synthesis such as thymidine block (causes cell cycle arrest at early S phase, where addition of excess thymidine causes a negative feedback loop that interrupts nucleotide biosynthesis). While these methods have led to a greater understanding of many cell cycle related processes, and indeed defined much of the somatic cell cycle, different methods of synchronization resulted in contradictory findings (Ballabeni et al., 2011). Also, as most of these methods are disruptive in nature, a common argument against their use is that they do not truly represent the natural state of the cells, and that off-target effects of drugs may have complicating phenotypes.

An example of contradictory results is seen in the early studies performed relating to the oscillatory behavior of Cyclin-Cdk activity in mES. Studies by Stead et al. and Fujii-Yamamoto et al. suggested that Cyclin A-Cdk2 and Cyclin E-Cdk2 do not show any oscillatory activity and their activity is sustained across the cell cycle (Stead et al., 2002; Fujii-Yamamoto et al., 2005). A later study by Ballabeni et al suggested the opposite, Cyclin-Cdk do show an oscillatory behavior across the cell cycle and suggested that the reason the earlier studies failed to detect it was due to “suboptimal synchrony” (Ballabeni et al., 2011). These differences could be attributed to the different methods used to synchronize cells: where the latter study used a double synchronization method to enrich for pure populations, the former used a single synchronization method. While both sets of studies were accurate based on their methods, the differences highlight one of the limitations of these techniques. An ideal system would be one in which it would be possible to monitor the behavior of individual cells in unperturbed populations.

### Using single cell imaging to study the ES cell cycle

The improvements in imaging techniques and associated analytical software have led to a host of newer methods which allows tracking and analysis of individual cells in an asynchronous population over long periods in a near native state. Overall, these advances have enhanced our understanding of cell cycle related process and sometimes led to revision of classical concepts. A study by Spencer et al shows that in somatic cells, the decision of whether or not to exit the cell cycle and enter quiescence is not taken in G1 as was previously thought but at a newly defined restriction point (Restriction Point 1) in the G2 phase of the preceding cell cycle which then modulates the levels of Cdk2 immediately after mitosis (Spencer et al., 2013). These observations challenge the long held belief that cell fate is decided at the RB regulated-“Restriction Point” in G1 (Pardee, 1974).

Using imaging techniques based on a Cdk2 sensor, this study showed that the levels of p21 at the Restriction Point 1 in the preceding cell cycle determined whether the cell will proliferate or enter quiescence in the subsequent cycle (Spencer et al., 2013). The use of the Cdk2 sensor permitted automated tracking of large numbers of individual cells through two or more cell cycles and permitted the establishment of a Cdk2-p21 threshold: subthreshold levels of p21 at the pre-M phase Restriction Point 1 led to a rapid increase in Cdk2 activity after mitosis, which permitted the cell to progress to the G1 phase (Spencer et al., 2013). High levels of p21 resulting in low Cdk2 activity post mitosis, resulted in cells that become transiently quiescent and, sensitive to mitogen withdrawal and are tunable to commit to the cell cycle until they pass the second (classical) Restriction point (Spencer et al., 2013). Thus, rather than a single point of integration of growth factor and nutirent availability in the RB-regualted G1/S checkpoint, the threshold of Cdk1 activity in the previous mitosis distinguishes two populations, which interpret the mitogenic environment in the subsequent G1 and determine cycling behavior.

In mES cells, due to the absence of p21, the Cdk2 sensor showed that mES cells are always predisposed to continue another round of replication (Spencer et al., 2013). This study could also explain why mES cells fail to enter quiescence during mitogen deprivation (serum starvation) (Schratt et al., 2001). The mechanism of cell cycle lengthening during differentiation of mES cells into distinct proliferative precursor lineages is not known, but it is possible that with gradual upregulation of p21 and subsequent increase in G1 length, the two Restriction points may begin to influence proliferation decisions.

### Using FUCCI to the study the ES cell cycle

By far the most useful recent technology developed to study the cell cycle is the Fluorescent Ubiquitination-based Cell-Cycle Indicator (FUCCI) system (Sakaue-Sawano et al., 2008). This sensor allows for live tracking and clear demarcation of cells in the G1and S/G2/M phase of the cell cycle and when combined with FACS, these subsets of cells can be further subdivided into Early G1, Late G1, S, and G2/M (Pauklin and Vallier, 2013; Singh et al., 2016). FUCCI has revolutionized how we study the cell cycle by creating a non-disruptive, near native system to study any cell cycle related phenomena. Further, the dual sensor-based system has expanded our repertoire to analyse changes in the cell cycle and cell cycle-related processes *in vivo* with the creation of model systems such as the FUCCI-Mouse, FUCCI-Zebrafish and FUCCI-Fly (Sakaue-Sawano et al., 2008; Sugiyama et al., 2009; Abe et al., 2013; Mort et al., 2014; Zielke et al., 2014).

The FUCCI system takes advantage of the periodic degradation of Geminin and Cdt1 that occurs during the cell cycle. Geminin is an inhibitor of replication origin firing that is expressed from S phase onward and degraded in M phase, while Cdt1 is required for replication origin licensing and accumulates from M to G1 phases, and is degraded at the onset of S phase. These two proteins are regulated by proteolysis to provide tight control of DNA replication. By fusing the degradation domains of Geminin and Cdt1 to different fluorescent reporters, it is possible to visually track cell cycle stages of the individual cells in an asynchronous population. Earlier, it was believed that Geminin, which is also important in maintaining pluripotency, was cell cycle independent in mES, unlike in somatic cells where it displays an oscillatory behavior (Fujii-Yamamoto et al., 2005; Gonzalez et al., 2006; Yang et al., 2011; Tabrizi et al., 2013). The FUCCI system permitted a re-evaluation of this notion, with the simple observation that Geminin/Cdt1-based oscillation was observable in mES cells transfected with FUCCI sensors indicates that Geminin activity is indeed cell cycle dependent and not independent as was shown earlier (Coronado et al., 2013). Use of the FUCCI system has also permitted increased reprogramming efficiency of somatic cells by selecting for cells with a shorter G1 (Roccio et al., 2013). These cells have a higher propensity to reprogram compared to cells with a longer G1, highlighting the link between a rapid cell cycle and the ability to attain pluripotency (Ruiz et al., 2011; Roccio et al., 2013; Guo et al., 2014).

By far the most divisive finding using the FUCCI system in mES is the study that found mES cells grown in “2i culture conditions” (presence of a MEK inhibitor PD0325901 and GSK3β inhibitor CHIR99021) actually have a lengthy G1 phase (Ter Huurne et al., 2017). This observation has upturned almost 30 years of thought that linked pluripotency with the shortened G1. The study showed that the reduced G1 seen in mES cells may be an adaptation to serum conditions due to increased ERK signaling. While this does raise the question of whether naïve ES cells actually have a longer G1 or not, *in vivo* analysis of embryo development post fertilization show a rapid expansion in the number of cells in the ICM before gastrulation with cell division times of ~10 h (Solter et al., 1971; Power and Tam, 1993). These contradictory results lead to a conundrum on whether the elongated cell cycle seen in 2i-grown mES cells truly represent what happens *in vivo* or represents another artifact of the culture system. Further work will be needed in this area to clearly mark out the differences seen between the *in vivo* and *in vitro* systems. Regardless, it is evident that unperturbed cell cycle studies can have a major impact on our interpretation of the regulatory system at the heart of cell division, and will probably cause re-evaluation or refinement of many control pathways in the near future.

### Using single cell RNA-seq to study the ES cell cycle

ES cell populations in culture exhibit substantial heterogeneity, with many key pluripotency factors such as Nanog displaying varying levels of expression, some of which can be attributed to cell cycle dependant control of protein abundance (Hatano et al., 2005; Singh et al., 2007; Gonzales et al., 2015; Liu Y. et al., 2017). While single cell imaging allows for greater resolution of heterogeneous populations, it is limited by the number of proteins that can be evaluated simultaneously. Using single cell qRT-PCR, it is possible to study a larger subset of genes but still limiting, especially while trying to address global alterations, or connections between networks of genes (Guo et al., 2010; Tang et al., 2010; White et al., 2011; Lecault et al., 2012).

Single cell RNA-Seq (scRNA-seq) is poised to make a major impact on analysis of global changes in gene expression as a read-out of cellular heterogeneity, but some technical challenges are yet to be overcome before it can be widely employed. These include the efficiency in parallel processing of a large number of cells, differentiating between technical noise and detectable low signal, sequencing depth for limiting material, and importantly, cost (Wu et al., 2014; Prakadan et al., 2017). Improvements in cell handling and better algorithms have increased the applicability of scRNA-seq to cell cycle studies (Trapnell et al., 2014; Macosko et al., 2015; Prakadan et al., 2017). For example, a recent algorithm uses scRNA-seq derived from single cells in asynchronous populations and is able to separate them into different cell cycle phases based on transcriptional signatures for each cell cycle phase (Santos et al., 2015; Scialdone et al., 2015).

Using scRNA-seq, genes encoding pluripotency factors, differentiation and cell cycle regulators have been found to be amongst the most variable in mES (Klein et al., 2015; Kolodziejczyk et al., 2015). Interestingly, cell cycle genes in mES showed a very weak transcriptional oscillation compared to somatic cells, suggesting that their cell cycle independent activity was also regulated at the transcriptional level (Klein et al., 2015). mES grown in serum could be divided into three subpopulations based on gene expression and cell cycle characteristics (Kolodziejczyk et al., 2015). While one subset expressed low or undetectable levels of Oct-3/4, Sox2, and Nanog, exhibited a slower cycling state and may be irreversibly committed to differentiation, a second subset expressing low levels of Nanog and high levels of Oct-3/4 and Sox2 was classified as an intermediate stage (Kolodziejczyk et al., 2015). The third and largest subset, classified as the self-renewing group expressed all the pluripotency factors at high levels accompanied by a relatively faster cell cycle (Kolodziejczyk et al., 2015). During differentiation triggered by Leukaemia Inhibitory Factor (LIF) withdrawal, most cells exhibited a rapid drop in Rex1 and Esrrb levels with the levels of Oct-3/4 and Sox2 dropping gradually, consistent with insights from bulk assays (Klein et al., 2015).

## Different states of pluripotency have different cell cycle patterns

During mouse embryonic development until gastrulation, cells range from a totipotent state to a multipotent state. Each of these progenitor cell types display distinctive epigenetic characteristics that regulate lineage determination and lead to unique functional properties (Soufi and Dalton, 2016). While pluripotent cells have been defined pre and post implantation, the remaining intermediate states have yet to be clearly mapped. This task is challenging due to the limited number of cells available *in vivo* and the difficulty in faithfully replicating these states *in vitro*. Preimplantation pluripotent cells are best represented by “Naïve” ES cells e.g., mES (Ying et al., 2008) while “Primed” ES cells such as mouse epiblast and human ES cells (hESC) are functionally equivalent to cells from the later epiblast stage of the post implantation blastocyst (Thomson et al., 1998; Brons et al., 2007; Tesar et al., 2007).

### Naïve ES cells

In mouse development, mES are derived from and phenotypically resemble cells from the ICM of pre implantation embryos of 3.5–4.5 days post coitum (dpc). Pluripotent cells from later stages of development such as 6.5 dpc can also be cultured *in vitro* and these can be converted to cells from earlier stages of development by either changing the culture medium, using small molecules that modulate key embryonic signaling pathways, or overexpression of transcription factors such as Klf4, highlighting the plasticity of these cell types at this stage (Bao et al., 2009; Guo et al., 2009; Zhou et al., 2010; Han et al., 2011). *In vitro*, mES cells are generally maintained in media containing serum + LIF and exhibit heterogeneous gene expression patterns. Subpopulations exhibit bimodal expression (High/Low) of many key pluripotency genes such as Nanog and Rex1 which are dynamic in nature, with many of these cells interchanging between High and Low states (Hatano et al., 2005; Singh et al., 2007; Han et al., 2010). Reports also indicate that these cells may exhibit gene profiles similar to endoderm as well as primed ES cells (Hayashi et al., 2008; Toyooka et al., 2008; Canham et al., 2010). While this fluidity of cell states poses challenges for studying the cell cycle, it is possible to isolate these modal populations for analysis. A good example is the isolation of mES cells expressing different levels of Nanog. Nanog^High^ cells express higher levels of many positive regulators of the cell cycle such as Cyclin B and E2F1 and show more rapid proliferation (Singh et al., 2007). By contrast, Nanog^Low^ cells express higher levels of Cdkns such as p21, p27 & p57 and cycle at a correspondingly slower rate (Singh et al., 2007; MacArthur et al., 2012). Thus, expression levels of the pluripotency factor Nanog preconfigures cell cycle rate in a population of mES.

The heterogeneity displayed by mES reflects a balancing act performed by the cells in maintenance of pluripotency in the presence of a combination of that trigger stemness (LIF) and differentiation (FGF) (Kunath et al., 2007). By culturing mES cells in serum-free media along with MEK inhibitor PD0325901 and a GSK3β inhibitor CHIR99021 (“2i” conditions), it is possible to minimize this heterogeneity leading to mES that are as close to naïve “ground state” as is currently possible *in vitro* (Ying et al., 2008). 2i conditions lead to higher expression of Nanog and a relatively faster proliferation rate (Marks et al., 2012) (Table 1). Though earlier reports did indicate that mES cultured in 2i expressed p21 yet showed a rapid proliferation rate (Marks et al., 2012) more recent results suggest that when compared to mES cultured in serum, 2i cells may actually proliferate relatively slower (Ter Huurne et al., 2017). The difference in the cell cycle lengths of mES cultured in serum vs. 2i conditions vary by a few hours, and even though there is a slowing of the cell cycle, the change is subtle, which may explain why earlier studies did not emphasize the issue.

**Table 1:** Comparing the activity and expression of key cell cycle genes in “naïve” mES cultured in serum free 2i conditions and “primed” mES cultured in serum containing media.

**Gene**	**Naïve mES**	**Primed mES**
Cyclin A	Expressed at high levels, not known if it is cell cycle independent or dependent	Expressed across the cell cycle
Cyclin B	Expressed only during G2/M	Expressed only during G2/M
Cyclin D	Expressed during G1	Low expression during highly reduced G1
Cyclin E	Expressed across the cell cycle	Expressed across the cell cycle
Cdk1	Expressed at high levels, not known if it is cell cycle independent or dependent	Expressed only during G2/M
Cdk2	Active across the cell cycle	Active across the cell cycle
Cdk4	Active during G1	Low expression
Cdk6	Active during G1	Low levels of activity during highly reduced G1
p16	Expressed during G1	No expression
p21	Expressed during G1	No expression
p27	Expressed during G1	No expression
RB	Active during G1	Inactive

### Primed ES cells

In contrast to mES, primed cells (cells derived from the epiblast of post implantation blastocyst) such as hESC show the presence of a functional G1 phase (Neganova et al., 2009; Pauklin and Vallier, 2013) (Figure 1A, Tables 2, 3). Primed ES cells express all the three D type cyclins, Cyclin D1, Cyclin D2, and Cyclin D3 and Cdk4 expression is higher than Cdk6 (in mES, Cdk6 is predominant) (Becker et al., 2006; Neganova et al., 2009). Unlike in mES, Cyclin E/Cdk2, and Cyclin A/Cdk2 activity is cell cycle dependent, and the RB checkpoint in G1 is functional (Becker et al., 2006; Ghule et al., 2007; Neganova et al., 2009). hESC also express low levels of p21 and p27, one of the key differences from mES and a major contributor toward the different cell cycle states (Neganova et al., 2009).

**Table 2:** A comparison of the expression and activity of some of the important positive cell cycle genes in mES and hESC.

**Gene**	**Function**	**Mouse embryonic stem cells**	**Human embryonic stem cells**	**References**
Cyclin A	Regulates S-phase with Cdk2	Expressed throughout the cell cycle	Expressed during late G1, S and G2	Stead et al., 2002; Ghule et al., 2007; Neganova et al., 2009
Cyclin B	Regulates M-phase with Cdk1	Expressed only during G2/M	Expressed only during G2/M	Stead et al., 2002; Ghule et al., 2007; Neganova et al., 2009
Cyclin D1	Regulates G1-phase with Cdk4 or Cdk6	Low expression during the highly reduced G1	Expressed during G1	Faast et al., 2004; Neganova et al., 2009
Cyclin D2	Regulates G1-phase with Cdk4 or Cdk6	No expression	Low expression	Faast et al., 2004; Pauklin and Vallier, 2013
Cyclin D3	Regulates G1-phase with Cdk4 or Cdk6	Low expression during the highly reduced G1	Expressed during G1	Faast et al., 2004; Neganova et al., 2009
Cyclin E	Regulates G1/S-phase with Cdk2	Expressed throughout the cell cycle	Expressed during late G1 and S	Stead et al., 2002; Ghule et al., 2007; Neganova et al., 2009
Cdk1	Regulates M-phase with Cyclin B	Maximal activity during G2/M	Maximal activity during G2/M	Stead et al., 2002; Ghule et al., 2007; Neganova et al., 2009
Cdk2	Regulates G1/S-phase with Cyclin E and S/G2 phase with Cyclin A	High activity throughout the cell cycle	High activity throughout the cell cycle	
Cdk4	Regulates G1-phase with Cyclin D1 or D2 or D3	Low expression	Maximal activity during G1	Faast et al., 2004; Neganova et al., 2009
Cdk6	Regulates G1-phase with Cyclin D1 or D2 or D3	Low levels of activity during the highly reduced G1	Maximal activity during G1	Faast et al., 2004; Neganova et al., 2009

**Table 3:** A comparison of the expression and activity of some of the important negative cell cycle genes in mES and hESC.

**Gene**	**Function**	**Mouse embryonic stem cells**	**Human embryonic stem cells**	**References**
p16	Inhibitor of Cyclin/Cdk activity	No expression	No expression	Faast et al., 2004; Zhang et al., 2009
p19	Inhibitor of Cyclin/Cdk activity	No expression	Low expression	Li et al., 2009; Zhang et al., 2009
p21	Inhibitor of Cyclin/Cdk activity	No expression	No/very low expression	Stead et al., 2002; Neganova et al., 2009
p27	Inhibitor of Cyclin/Cdk activity	No expression	No/very low expression	Stead et al., 2002; Egozi et al., 2007; Neganova et al., 2009
p57	Inhibitor of Cyclin/Cdk activity	No expression	No expression	Becker et al., 2006; Sorrentino et al., 2007
Rb	Maintains the Restriction point in G1	Inactive	Active during G1	Savatier et al., 1994; Conklin and Sage, 2009

The cell cycle of mouse epiblast derived stem cells (EpiSC) is not as well characterized as mES or hESC but as they are developmentally equivalent to hESC, one can speculate that they exhibit a similar cell cycle profile to hESC (Brons et al., 2007; Tesar et al., 2007). When compared to mES, EpiSC express similar levels of Oct-3/4 but lower levels of Nanog (Guo et al., 2009; Han et al., 2010) which may suggest a cell cycle profile similar to Nanog^Low^ mES (Singh et al., 2007; MacArthur et al., 2012). EpiSC also express many differentiation markers such as Brachyury, Eomes, Sox17 & Gata6, characteristic of later development stages closer to gastrulation (Kojima et al., 2014). A detailed analysis of the EpiSC cell cycle could be very informative about how fate determination influences cell cycle changes and the role of lineage determinants in these pathways.

A recent development by Yang et al. showed it was possible to culture pluripotent cells of human and mouse origin which can contribute to both the embryonic and as well as extraembryonic tissue (Yang et al., 2017). The study used a serum free culture system which consists of a chemical cocktail containing human LIF (hLIF), Gsk3β inhibitor CHIR 99021, (S)-(+)-dimethindene maleate (DiM) which targets G protein coupled receptors and minocycline hydrochloride (MiH) which inhibits Parp1. The resultant cells were termed as Extended Pluripotent Stem cells (EPS), and appear to be developmentally equivalent to the zygote or early blastomeres as they can rise to both embryonic as well as extra embryonic tissue (Tarkowski, 1959). This discovery when combined with the other pluripotent stages captured *in vitro* and, technologies such as FUCCI and single cell RNA-Seq that can yield information about individual cells with a population, opens up exciting new possibilities in studying embryonic development with respect to the cell cycle and how cell fate may alter the cell cycle during embryogenesis.

## Setting the right time for differentiation

The classical view with regards to differentiation suggests that embryonic stem cells are receptive to differentiation cues during the G1 phase when lineage determination is decided. This concept is similar to the “Restriction point” in G1 phase, which is marked as the point during G1 in which a cells decides on whether to continue to proliferate or enter quiescence (Pardee, 1974). Yet, with mES cells exhibiting a highly reduced, almost non-existent G1 phase, the timing of differentiation decisions is more difficult to establish.

### Differentiation during G1 phase

Studies using the FUCCI system have been able to isolate mES cells specifically during the shortened G1 phase and show that they are more susceptible to differentiation cues (Retinoic acid) than cells in S phase or G2-phase (Coronado et al., 2013). Also as cells move from a naïve state to a primed state as defined by the expression levels of the transcription Rex1, there is a measurable increase in the length of G1 phase (Coronado et al., 2013). This increased G1 length leads to activation of RB along with increased susceptibility to differentiation cues (Coronado et al., 2013). Rex1, like Nanog, exhibits a bimodal expression in cultured mES, where Rex1^High^ mES cells are thought to represent cells from the ICM (naïve) and Rex1^Low^ cells are considered developmentally equivalent to Epiblast or Primitive Ectoderm (primed) (Toyooka et al., 2008).

By contrast, hESC exhibit a clear G1 phase accompanied by Cyclin D expression as well as an active RB (Becker et al., 2006; Ghule et al., 2007; Neganova et al., 2009; Pauklin and Vallier, 2013). Sorting hESC into their corresponding cell cycle phases using centrifugal elutriation has also shown that similar to mES, hESC have a greater propensity to differentiate or are more receptive to differentiation cues during G1 phase compared to S phase or G2 (Sela et al., 2012). By blocking the activity of SRC non-receptor tyrosine kinase in hESC, it was possible to keep RB in an unphosphorylated/hypo-phosphorylated active state, thereby extending G1 and leading to increased efficiency of differentiation potential (Chetty et al., 2015). Utilising the FUCCI system, it was shown that based on the period of time spent in G1, hESC were biased toward a particular lineage (Pauklin and Vallier, 2013). Sorted sub-sets of G1 cells that were plated into defined culture conditions had a higher propensity to differentiate into endoderm or mesoderm when shifted in early G1 phase, but into neuro-ectoderm during the late G1 phase (Pauklin and Vallier, 2013).

One mechanism by which hESC cells might be receptive to differentiation signals during G1 phase is through the expression of the three D type cyclins, namely D1, D2, and D3 (Neganova et al., 2009; Pauklin and Vallier, 2013). During early G1 when Cyclin D levels are low, Smad2/3 is free to bind to and activate endodermal genes, facilitating differentiation toward endodermal lineage (Pauklin and Vallier, 2013; Pauklin et al., 2016). As G1 progresses, Cyclin D expression is induced and represses endodermal genes, preventing differentiation toward that fate (Pauklin et al., 2016). The levels of Cyclin D during mid and late G1 determine the propensity to differentiate toward either mesoderm or neuro-ectoderm (Pauklin and Vallier, 2013; Pauklin et al., 2016). Upregulation of Cyclin D2 and mild increase in Cyclin D1 and Cyclin D3 expression correlated with mesodermal differentiation, while upregulation of all three D type cyclins led a neuro-ectoderm fate (Pauklin and Vallier, 2013; Pauklin et al., 2016).

While knockdown of any of the D type Cyclins individually did not affect hESC differentiation toward any particular lineage, double knockdowns showed a propensity to differentiate into endoderm/mesoderm and a reduced capacity to differentiate into neuro-ectoderm (Pauklin and Vallier, 2013; Pauklin et al., 2016). Triple knockdown hESC could not be propagated, suggesting a crucial function in hESC survival and/or self-renewal, which is in contrast to triple Cyclin D knockout mES cells which show no noticeable phenotype (Pauklin and Vallier, 2013; Huskey et al., 2015; Liu L. et al., 2017). These differences in requirement for Cyclin D might reflect the different developmental stages hESC and mES are thought to represent. Also, as mES cells have a reduced G1, the mES cell cycle may have adapted to the low levels of Cyclin D. This would tie in well with the fact that two well-known functions of Cyclin D are, to act as a sensor for mitogenic signals and phosphorylation of RB, which are both probably suppressed to prevent differentiation in mES (Sherr and Roberts, 1995).

A second potential mechanism explaining the responsiveness of ES cells to differentiation cues during G1 might reflect the transient epigenetic change in “bivalent domains” of many developmental genes (Bernstein et al., 2006; Singh et al., 2013). In ES cells, many lineage specifying genes are dually marked (“bivalent”) by large regions of the repressive H3K27me3 mark, overlapping shorter regions of the activating H3K4me3 mark. This bivalent status correlates with a poised state that is resolved toward a single predominant mark during commitment and lineage transition (Bernstein et al., 2006) As ES cells exit pluripotency and enter differentiation, there is a rapid loss of the repressive H3K27me3 mark at these sites. Interestingly in hESC, a few developmental genes such as Sox17 and Gata6 express at low levels during the G1 phase (Singh et al., 2013). While this “leaky” expression does not lead to overt differentiation, it could create a “window of opportunity” during the G1 phase, rendering ES cells more susceptible to differentiation.

### Is the shortened G1 phase crucial to prevent differentiation?

The reduced G1 phase is considered to be an intrinsic characteristic of pluripotent stem cells, and has been proposed to reduce their susceptibility to differentiation. First described more than 30 years ago this idea has been the prevalent viewpoint in the field (Mummery et al., 1987). However, several studies have since challenged this notion. By overexpressing either p21 or p27, two potent Cdk inhibitors, it is possible to elongate the length of G1 phase in mES cells (Li V. C. et al., 2012). Using this perturbation, it was shown that an elongated G1 phase in mES did not lead to a reduction in the levels of Oct-3/4, Nanog or SSEA-1 in the basal state, nor did it lead to increased differentiation potential during LIF withdrawal (Li V. C. et al., 2012). However, overexpression of Cyclin E or Cyclin A in mES did lead to delayed differentiation as based on Nanog expression as a readout for pluripotency (Li V. C. et al., 2012). A similar result was obtained in hESC where overexpression of p21 did not lead to increased differentiation during LIF withdrawal (Gonzales et al., 2015). In contrast, overexpressing p27 in hESC had the opposite effect, where it led to a G1 arrest but no significant changes in pluripotency markers (Menchón et al., 2011). Interestingly, loss of p27 in hESC led to upregulation of Brachyury and Twist, demonstrating a novel function for p27 in maintaining pluripotency (Menchón et al., 2011). The notion of a shortened cell cycle leading to conditions unfavorable for differentiation is further challenged by the recent finding that naïve mES (grown in 2i conditions) exhibit a longer G1 than mES grown in normal serum conditions (Ter Huurne et al., 2017).

Thus, while the evidence in favor of G1 duration as a key determinant of differentiation is not definitive, it is possible that more targeted experimental perturbations may be informative. For example, extending the G1 phase of ES cells using an inducible p21 or p27 system might reveal that ES cells can tolerate a certain level of p21/p27 expression and that subsequent extension of G1 phase could tilt the balance to differentiation. Also, by gradually increasing the G1 phase, this system might mimic the gradual increase seen in the G1 phase length as pluripotent cells differentiate during embryogenesis (Mac Auley et al., 1993; Lange and Calegari, 2010).

### Differentiation in other phases of the cell cycle

Are ES cells refractory to differentiation cues in the other phases of the cell cycle? Earlier studies have concluded that hESC in S or G2 phase are less susceptible to differentiation cues compared to those in G1 (Pauklin and Vallier, 2013). Indeed, elongating either S phase or G2 phase actually enhances pluripotency (Gonzales et al., 2015). Using genetic and chemical perturbations, this study shows that by prolonging the duration of S phase or delaying mitotic transition (increased G2 phase), hESC take longer to differentiate on withdrawal of LIF (Gonzales et al., 2015). There was no accompanying change in the length of G1 during this process, thereby indicating that elongating S or G2 did not reduce the accessibility for differentiation cues by reducing the length of G1 (Gonzales et al., 2015).

## ES cells have modified cell cycle checkpoints

A cycling cell has checkpoints interspersed throughout the cell cycle that monitor a variety of cell signals and function as a brake, preventing progression to the next phase until certain criteria are met. These checkpoints are crucial in ensuring that key conditions such as optimal external mitogenic conditions (Restriction point), faithful DNA replication (S phase checkpoint), preservation of genomic integrity (DNA damage checkpoint), and proper chromosome segregation (spindle assembly checkpoint) are satisfactorily completed before transitioning to the next phase. A disabled or faulty checkpoint results in aberrant or precocious transitions and can eventually lead to cell death or malignant transformation. In ES cells, these checkpoints are either absent or are modified to cater to the rapid cell cycle.

### RB's duality

RB has traditionally been considered as the chief gatekeeper of the G1 Restriction point which prevents cells from entering S phase (Pardee, 1989; Weinberg, 1995). This control is primarily brought about by modifying the phosphorylation status of RB. When a cell enters G1, RB exists in an active (unphosphorylated) state and blocks transcription of key G1/S phase genes, preventing passage across the Restriction point (Weinberg, 1995; Lundberg and Weinberg, 1998). Phosphorylation of RB across G1 reduces its inhibitory activity, and induces a series of reactions which inactivate the Restriction Point and permit S phase entry (Weinberg, 1995; Lundberg and Weinberg, 1998). Once the Restriction Point has been crossed, cells become independent of extrinsic mitogenic signals. In mES cells grown in serum, RB is in a perpetually hyperphosphorylated form which allows for a rapid transition from M to S phase, essentially bypassing G1 (Savatier et al., 1994; Ter Huurne et al., 2017). As the G1 phase in hESC is relatively longer, they show the presence of both an active and inactive form of RB (Filipczyk et al., 2007; Conklin et al., 2012), mES grown in 2i conditions also display a similar profile (Ter Huurne et al., 2017).

While RB's role as a cell cycle checkpoint is clear, this tumor suppressor plays different roles in mES and hESC. Compromising RB expression in either mES or hESC respectively resulted in increased genomic instability, yet mES continue to proliferate while hESC do not (Dannenberg, 2000; Sage, 2000; Zheng et al., 2002; Conklin et al., 2012). In contrast, triple knockout (TKO) mutants of the pocket proteins (RB, p107, p130) resulted in different phenotypes for mES and hESC. While mES cells did not show any noticeable phenotype and proliferated normally, hESC showed an increase in the levels of p21, arrested at G2/M and displayed increased cell death (Conklin et al., 2012). It is not clear why mES and hESC display different phenotypes, though one possibility is that since mES and hESC represent different stages of development, the RB family may play different roles in self-renewal and proliferation at different stages of development. Further analysis is required to establish and define the mechanisms by which developmental stage may influence restriction point control.

### p53–guardian of pluripotency

The tumor suppressor p53 is a pivotal regulator of genome stability. In case of DNA damage, two major signaling cascades prevent cells from proceeding to the next cell cycle phase. These are the ATM-Chk1 and the ATR-Chk2 signaling cascades (Sancar et al., 2004; Smith et al., 2010) which are activated in response to DNA double-strand breaks (DSB) and single stranded DNA (SSD) respectively. Both ATR and ATM directly activate p53 by phosphorylation and indirectly via Chk1 and Chk2 kinase activity (Banin et al., 1998; Canman et al., 1998; Chehab et al., 1999). The change in phosphorylation status inhibits nuclear export of p53, leading to its accumulation in the nucleus (Zhang and Xiong, 2001). There, p53 activates p21 expression which binds to and inhibits Cyclin E/Cdk2 and Cyclin A/Cdk2 activity, creating a G1/S phase block (El-Deiry et al., 1993; Harper et al., 1993).

In ES cells, the low levels of p21 and high levels of Cdk2, negate the DNA damage -induced slowing of the cell cycle seen in somatic cells (Aladjem et al., 1998; Chao et al., 2000; Hong and Stambrook, 2004). Instead, there is a greater propensity for apoptosis during genomic stress in ES cells. p53's role as an inducer of apoptosis in response to DNA damage in somatic cells is well-known, yet its functioning in mES is contested. Earlier studies show that while DNA damage causes apoptosis in mES, the dependence on p53 is a matter of Aladjem et al. (1998), de Vries et al. (2002), Solozobova et al. (2009), and van der Laan et al. (2013). New studies suggest a p53-dependent role in apoptosis at least for doxorubicin-induced DNA damage (Li et al., 2015; He et al., 2016).

In hESC, the link between UV-induced DNA damage and p53-induced apoptosis is well characterized, though apoptosis is induced through the mitochondrial pathway rather than a direct activation of apoptotic genes (Qin et al., 2007). Also, hESC cells have been shown to express more pro-apoptotic genes compared to anti-apoptotic genes after DNA damage (Dumitru et al., 2012; Li M. et al., 2012; Liu J. C. et al., 2013) tilting the balance toward cell death.

p53 plays a unique role in ES cells where it functions as a guardian of pluripotency in response to DNA damage, a unique adaptation to preserve genome integrity in these cells which will give rise to all cells including the germline. When p53 is activated during DNA damage, it suppresses expression of Oct-3/4 and Nanog in hESC and Nanog in mES by directly binding and repressing their promoters, leading to differentiation (Lin et al., 2005; Qin et al., 2007). As ES cells are the precursors for all tissues (except extra-embryonic) formed during embryogenesis, it is imperative that any DNA damage not be propagated to the daughter cells. By causing differentiation, p53 in effect prevents the propagation of DNA damage. This process can be mimicked in hESC by using Nutlin, which activates the p53 DNA damage response cascade in the absence of DNA damage (Maimets et al., 2008). Interestingly, there is p53-dependent upregulation of RB during DNA damage response in mES, suggesting that p53 might be induce differentiation via RB (He et al., 2016).

### Modified DNA damage response

ES cells are known to be hypersensitive to DNA damage: as progenitors to all tissues including the germline, it is imperative to minimize mutations passed onto the next generation. However, important G1/S, S phase and G2/M phase checkpoints are missing in ES cells, yet they still preserve genomic integrity. How do ES cells achieve this?

A major source of mutations in genomic DNA is due to reactive oxygen species (ROS) produced as by-products of oxidative phosphorylation. ES have evolved many unique mechanisms to protect their genome from ROS-mediated mutations. Firstly, ES cells primarily use glycolysis for energy production which while less energy efficient, produces lower ROS than oxidative phosphorylation (Kondoh et al., 2007; Folmes et al., 2011; Xu et al., 2013). This helps them maintain lower levels of ROS compared to differentiated cells (Saretzki et al., 2004, 2008; Cho et al., 2006). mES also express higher levels of antioxidant genes such as Sod2 which is downregulated during differentiation (Saretzki et al., 2004). Further, ES cells have fewer mitochondria that are less active and contain poorly developed cristae (St. John et al., 2006; Facucho-Oliveira and St. John, 2009; Prigione et al., 2010). All of these mechanisms combine to create an environment that reduces ROS generation and the possibility of ROS induced mutations.

During double strand breaks, ES cells exhibit higher levels of ATM and ATR dependent phosphorylation of γH2AX, which amplifies the DNA double strand break damage response (Shiloh, 2003). In hESC, ATM and ATR both phosphorylate γH2AX, thereby increasing the efficiency of repair (Shiloh, 2003; Adams et al., 2010). ES cells also express higher levels of DNA repair genes (Momcilovic et al., 2010; Tichy et al., 2010), leading to a more efficient and rapid DNA damage response than their somatic counterparts. Further, ES cells preferentially use homologs recombination to repair double strand breaks leading to better fidelity compared to somatic cells, where the error prone non-homologs end joining pathway is dominant (Momcilovic et al., 2010; Tichy et al., 2010).

Overall, in order to maintain the rapid proliferation, the cell cycle checkpoints in pluripotent stem cells have been modified to perform additional tasks along with their general cell cycle regulatory functions. RB and p53 can initiate the differentiation process, a role unique to ES cells, highlighting the interdependence of pluripotent state and the cell cycle.

## Mitotic bookmarking by pluripotency factors

During mitosis, along with chromosome compaction, there is a general decrease in gene expression associated with depletion of many transcription factors (TFs) from their target binding sites (Taylor, 1960; Prescott and Bender, 1962; Johnson, 1965; Martínez-Balbás et al., 1995). Chromatin re-association of TFs and subsequent gene activation resumes after entry into G1 (Taylor, 1960; Prescott and Bender, 1962; Johnson, 1965). Since ES cells shuttle rapidly between M phase and S phase, the rapid chromatin association of pluripotency factors would be necessary to maintain their ability to self-renew.

To faithfully re-establish stem cell expression states following M phase, several mechanisms have been proposed to occur that are heritable through the condensation-decondensation dynamics of mitotic chromosomes, including DNA methylation patterns, stable histone modifications and bookmarking by transcription factors (Kadauke and Blobel, 2013). TFs that remain bound to chromatin during mitosis are considered to “bookmark” the chromatin such that re-association occurs rapidly to quickly re-establish expression of genes post mitosis (Michelotti et al., 1997). Many of the TFs identified that bookmark chromatin are either master regulators of cell lineage such as GATA1 in the case of erythroid development or “pioneer” factors such as FoxA1 which can access inaccessible nucleosome positions during liver development (Kadauke et al., 2012; Caravaca et al., 2013).

In ES cells, Esrrb, Sox2, Oct-3/4, and Klf4 have been shown to bookmark chromatin during mitosis (Deluz et al., 2016; Festuccia et al., 2016; Teves et al., 2016; Liu Y. et al., 2017). Interestingly, the role of many major TFs in mitotic bookmarking may have been overlooked due to a fixation artifact that occurs during immunostaining (Teves et al., 2016). The use of paraformaldehyde as a fixative causes a depletion of transcription factors along the chromatin during mitosis, which probably delayed the discovery that pluripotency factors act to bookmark mitotic chromatin (Teves et al., 2016). Earlier studies indicated that Oct-3/4 does not bookmark chromatin (Galonska et al., 2014) which was later disproved using live cell imaging (Liu Y. et al., 2017).

In mES, using a fusion construct that is degraded at the M-G1 transition, persistent chromatin association of Oct-3/4 and Sox2 was found to be important for maintaining the pluripotent state, as the loss of these factors specifically during M phase led to increased differentiation (Deluz et al., 2016; Liu Y. et al., 2017). Interestingly, when the same constructs were used for reprogramming, it was found that degradable Oct-3/4 in combination with the other Yamanaka factors could not reprogram MEFs, while degradable Sox2 could. The biological significance of this difference is not clear, but it is conceivable that there is a greater susceptibility to reprogram the genome in M phase, as many lineage specific transcription factors are expelled from chromatin and the global transcription rate is low (Egli et al., 2007; Ganier et al., 2011; Halley-Stott et al., 2014).

## Oct-3/4 is a master cell cycle regulator in ES

**Figure 2:**
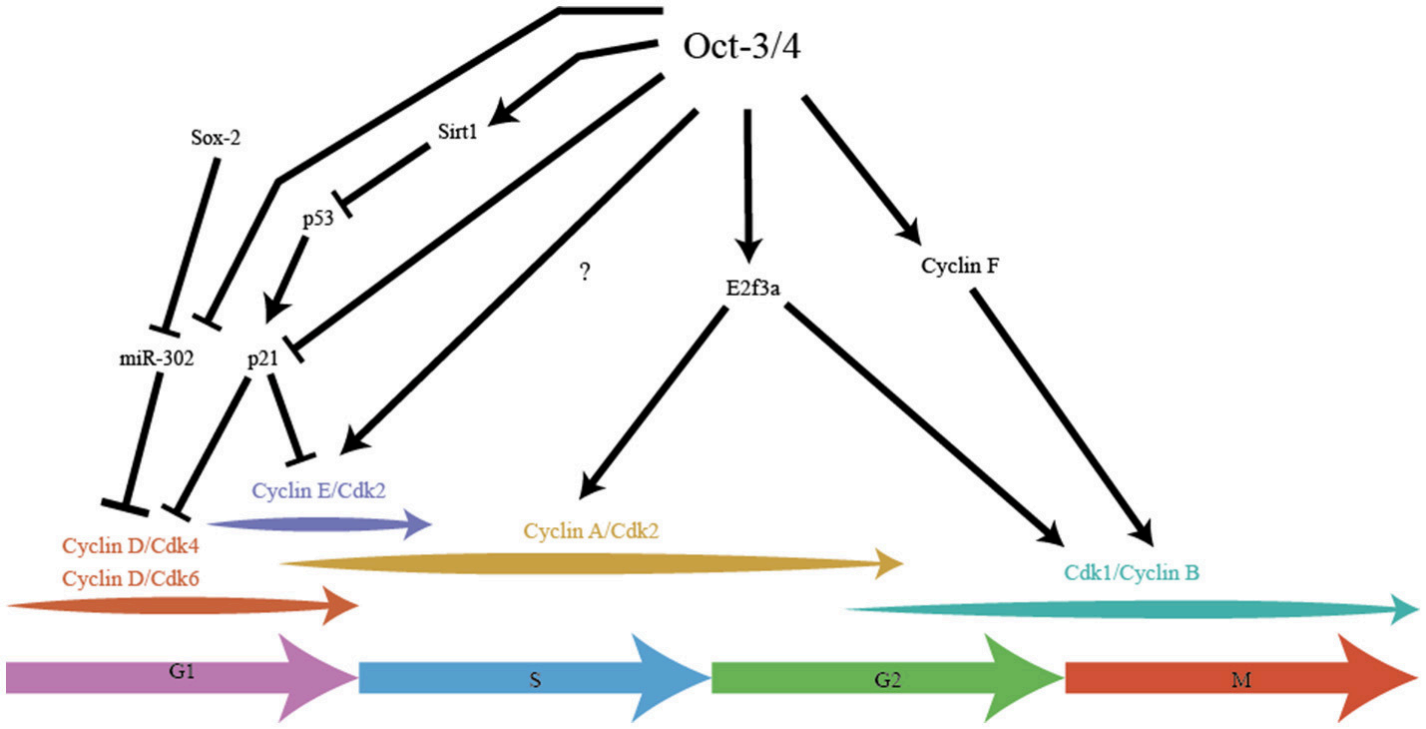
Pluripotency factor Oct-3/4 integrates stemness with cell cycle in cell cycle speed. Oct-3/4 plays an important role in maintaining the different phases of the cell cycle in ES cells. Oct-3/4 in collaboration with Sox-2, regulates Cyclin D/Cdk activity via miR-302, ensuring a shorter G1. Oct-3/4 represses p21 activity by directly inhibiting its expression and indirectly, by inhibiting p53, a potent activator of p21 expression. Oct-3/4 positively regulates expression of E2F3a which is the main E2F activator for Cyclin A and Cdk1 expression. Oct-3/4 positively regulates Cyclin F which aids in the migration of Cyclin B into the cell nucleus, thereby promoting G2/M. The arrows indicate positive regulation, the T-line represents inhibition.

Oct-3/4 was one of the earliest pluripotency factors discovered and arguably one of the most important (Schöler et al., 1989; Okamoto et al., 1990; Rosner et al., 1990). Knockout studies of Oct-3/4 show that the embryo develops to the blastocyst stage, but the ICM does not contain pluripotent cells, instead ICM cells are primed to differentiate to the extraembryonic trophoblast lineage (Nichols et al., 1998). The levels of Oct-3/4expression is extremely tightly regulated, as any deviation results in differentiation of ES cells (Niwa et al., 2000). While this highlights the importance of Oct-3/4 in maintaining pluripotency, this TF also plays a crucial role in maintaining many key aspects of the modified ES cell cycle (Figure 2).

In hESC, Oct-3/4 along with Sox-2 regulates the transcription of the miR-302 cluster which reduces expression of Cyclin D1 and shortens G1 phase (Card et al., 2008). Knockdown of the miR-302 cluster in hESC leads to an increase in the frequency of cells with extended G1, highlighting the role of these miRNAs in maintaining the pluripotent cell cycle. The miR-302 cluster is expressed 6.5 dpc to 8.5 dpc but not at 3.5 dpc (Card et al., 2008) which is consistent with the embryonic developmental stages that hESC represent.

High levels of Cyclin E have been associated with high levels of Oct-3/4 in head and neck squamous carcinoma cells (HNSC), leading to the use of Oct-3/4 as prognostic marker for this cancer (Koo et al., 2015). HNSC that expressed high levels of Oct-3/4 exhibited enhanced stem cells traits, better self-renewal and greater proliferation. Knocking down Oct-3/4 in HNSC led to a suppression of HNSC stem cell like properties (Koo et al., 2015). These findings are consistent with overexpression of Cyclin E in ES which show high Oct-3/4 expression, greater pluripotency and reduced differentiation potential (Coronado et al., 2013; Krivega et al., 2015). Although not explicitly demonstrated, it is quite likely that Oct-3/4 could directly regulate Cyclin E in ES cells, thus influencing the rapid G1-S transition.

Oct-3/4 represses the activity of p53 by regulating the expression of Sirt1, a protein deacetylase that inactivates p53 in hESC (Zhang et al., 2014). During retinoic acid-induced differentiation or knock down of Oct-3/4, Sirt1 is also repressed, leading to increased p53 activity, increased expression of differentiation genes and down-regulation of pluripotency genes (Zhang et al., 2014). Absence of deacetylation by Sirt1 leads to stabilization of p53 protein and subsequent increased p53 activity (Zhang et al., 2014). Overexpressing Sirt1 in an Oct-3/4 knockdown background leads to a reversal of the phenotype (Zhang et al., 2014). Oct-3/4 also directly represses the expression of p21, the downstream effector of p53 in mES (Lee et al., 2010). Since expression of p21 leads to an increase in the length of the G1, Oct-3/4 is ensuring the short G1 seen in mES by repressing p21. Whether Oct-3/4 regulates the low p21 expression seen in hESC is not known.

In mES, Oct-3/4 has been shown to positively regulate E2F3a expression and overexpression of E2F3a leads to faster proliferation in mES with low levels of Oct-3/4 (Kanai et al., 2015). It has been suggested that E2F3 is the main E2F regulating the transcription of B-Myb, Cyclin A, Cdk1 and Cdc6 (Humbert et al., 2000). E2F3 has also been shown to represses p19 by directly binding to the promoter and repressing transcription (Aslanian et al., 2004; Danielian et al., 2008). This is clearly manifested in E2F3 null MEFs that show a reduced proliferation rate (Humbert et al., 2000). A potential mechanism for the higher proliferation rate could be due to E2F3's ability to repress p19.

Oct-3/4 also plays a role in regulating mitosis. Cyclin B is mainly found in the cytoplasm during interphase and migrates to the nucleus during G2/M by forming a complex with Cyclin F (a non-canonical Cyclin that functions without a Cdk partner and ubiquitinylates rather than phosphorylates target substrates) (Kong et al., 2000; D'angiolella et al., 2013). Oct-3/4 has been shown to positively regulate Cyclin F (Campbell et al., 2007) in mES which could indirectly control the amount of Cyclin B thereby promoting G2/M phase entry. Oct-3/4 also plays an unusual role by indirectly inhibiting the activity of Cdk1. Oct-3/4 binds to Cdc25c, counteracting its function in removing the inhibitory phosphate marks on Cdk1, thereby preventing Cyclin B-Cdk1 complex formation (Zhao et al., 2014). The inhibitory effect of Oct-3/4 is mild and might represent one of the many thresholds present in mES that prevent premature entry into mitosis. In a contrasting role, Oct-3/4 also prevents differentiation of mES cells with the help of Cdk1. Cdk1 binds with Oct-3/4 to represses transcription of Cdx2 (Li L. et al., 2012) a master regulator of the trophectoderm lineage (Niwa et al., 2005). This repression is cell cycle-independent and may underlie the failure of Cdk1 null embryos to develop to the morula and blastocyst stage (Santamaria et al., 2007).

Finally, Oct-3/4 regulates the activity of protein phosphatase 1 (PP-1) in mES by positively regulating the expression of Nipp1 (PPP1R8) and Cyclin F (Campbell et al., 2007). Nipp1 and Cyclin F both repress the activity of PP1 and by ensuring that RB is not dephosphorylated, Oct-3/4 ensures that RB is inactivated throughout the cell cycle. Thus, the pluripotency factor Oct-3/4 impacts the cell cycle at multiple points, contributing to the rapid proliferative rate.

## The role of Nanog in regulating ES cell cycle

Compared to Oct3/4, less is known about other pluripotency factors in regulating the ES cell cycle. Overexpression of Nanog in hESCs leads to faster proliferation rates with a reduced G1 (Zhang et al., 2009). There is also an increase in the levels of CDC25A and CDK6 during Nanog overexpression (Zhang et al., 2009). Interestingly, unlike Oct-3/4, the amount of Nanog protein fluctuates during the cell cycle, with the highest amounts being found during G1 (Gonzales et al., 2015; Liu Y. et al., 2017). As CDC25A regulates the entry of the cells in to S phase by activating CDK2, this suggests that Nanog might play a role in regulating the G1-S transition in ES cells (Hoffmann et al., 1994; Blomberg and Hoffmann, 1999; Zhang et al., 2009). These observations gain further biological significance as Nanog is expressed in many cancer cells, and knockdown leads to G1 arrest (Han et al., 2012; Cao et al., 2013; Jeter et al., 2015).

## Cell cycle genes directly control pluripotency

The key genes regulating the cell cycle also directly regulate pluripotency. Many studies have shown the influence of these genes in maintaining pluripotency and how it is possible to create a pluripotent like state by manipulating these genes. This is probably best exemplified in the complete reprogramming of somatic cells to an induced pluripotent state. Using the concept that a faster cell cycle is a key component of pluripotency, it is possible to drastically increase the efficiency of reprogramming.

### Positive cell cycle regulators that control pluripotency

Overexpression of Cyclin D1 along with its Cdk partner, Cdk4 results in a 10 fold increase in reprogramming efficiency in BJ fibroblasts which normally exhibit low reprogramming efficiencies (Ruiz et al., 2011). Knockdown of either Cdk1 or Cdk2 causes mES cells to spontaneously differentiate (Stead et al., 2002; Zhang et al., 2011; Huskey et al., 2015) highlighting a potential role in maintaining pluripotency. Cdk2 has been shown to phosphorylate Sox2 at S39 and S253, which while not important to maintain pluripotency in mES, is essential for reprogramming MEFs (Ouyang et al., 2015). The function of this phosphorylation is not known, but it might play a role in altered Sox2 degradation during the reprogramming process.

Knockouts of both Cyclin E1/E2 in mES show no significant changes in proliferation rates (Geng et al., 2003; Parisi et al., 2003; Huskey et al., 2015; Liu L. et al., 2017). Further, mES cells bearing quintuple knock out of the G1 Cyclins (all isoforms of Cyclin D and Cyclin E) cells can proliferate; albeit at a slightly reduced rate suggesting a non-essential role of Cyclin E for mES proliferation (Liu L. et al., 2017). On the other hand, knockdown of Cyclin E in human fibroblasts (hFib) completely abrogated iPS formation (Ruiz et al., 2011) and quintuple knock out of Cyclin D and Cyclin E isoforms in mES cells show reduced levels of Oct-3/4, Sox2 and Nanog protein but unchanged transcript levels (Liu L. et al., 2017). Targeted removal of either of the G1 Cyclins in mES led to increased expression of the alternative G1 Cyclin while maintaining the protein levels of Oct-3/4, Sox2 and Nanog, suggesting a compensatory mechanism (Liu L. et al., 2017). With mES cells expressing low levels of Cyclin D (Faast et al., 2004) and high levels of Cyclin E (Stead et al., 2002) these features all point to a role for Cyclin E in maintaining the pluripotency factor expression. Thus, while Cyclin E does not play a crucial a role in maintaining the high proliferation rates seen in mES, it is important for maintaining the pluripotent state.

Cyclin A is crucial for proliferation in mES cells, as targeted inactivation leads to cell cycle arrest even in the presence of Cyclin E (Kalaszczynska et al., 2009). One possible explanations is that mES cells are more dependent on Cyclin A than Cyclin E, as the levels of Cyclin A are higher than Cyclin E (Kalaszczynska et al., 2009). This notion is corroborated by phenotype of quintuple knockout of all G1 cyclins in mES, which showed little effect on cell proliferation and little change in the levels of Cyclin A (Liu L. et al., 2017). A second possibility concerns B-Myb which is transactivated by the kinase activity of Cyclin A/Cdk2 but not Cyclin E/Cdk2 (Robinson et al., 1996; Saville and Watson, 1998). B-Myb is crucial for G1/S phase transition, and knockdown of B-Myb in mES cells also leads to aneuploidy, along with stalling at G2/M (Zhan et al., 2012). Further, B-Myb has been shown to bind to and positively regulate Sox2 and Nanog promoters, while its own promoter has binding sites for Oct-3/4, Sox2, and Nanog (Zhan et al., 2012), suggesting a positive feedback loop. The importance of B-Myb in maintaining the pluripotent state is evident, but whether it is Cyclin A/Cdk2 dependent is not known.

### Negative cell cycle regulators that control pluripotency

RB's role as the gatekeeper of the Restriction Point is well-known, but in ES cells, RB also maintains the pluripotent state. During mES differentiation, there is an enrichment of RB/E2F at the promoters of Oct-3/4 and Sox2, along with a subsequent decrease in their expression levels (Kareta et al., 2015a). This repression is partially removed in RB null MEFs which express low but significant levels of Oct-3/4 and Sox2 without affecting their proliferation rates (Kareta et al., 2015a; Vilas et al., 2015). RB null MEFs can be reprogrammed more rapidly and efficiently to iPS with all four of the Yamanaka factors (Kareta et al., 2015a), as well as with just two factors (Oct-3/4 and Klf4) albeit with lower efficiency and a longer reprogramming duration (Vilas et al., 2015).

The tumor suppressor Ink4/Arf also has an important role in reprogramming. In MEFs, it is the p19 product of that locus which is the major barrier to reprogramming while in human fibroblasts, p16 is the major hurdle (Li et al., 2009). Knocking out p19 in MEFs leads to faster reprogramming while knocking out p16 has no noticeable effect, with the opposite being the case in human cells (Li et al., 2009). These findings suggest differences in the path taken by human and mouse fibroblasts toward reprogramming, which might reflect species differences in regulatory programs between mES and hESC. Low levels of the Cdkn p57 appear to confer an “elite” status to cells, allowing them to reprogram faster but the mechanism is not known (Guo et al., 2014).

During mES differentiation, p21 and p27 both repress expression of Sox2 by binding to the Sox2 Regulatory Region 2 (SRR2) enhancer (Li H. et al., 2012; Yamamizu et al., 2014). p27 binds along with the p130-Ef24-Sin3a repressor complex to the SRR2 to repress Sox2 during differentiation (Li H. et al., 2012). Whether p21 also binds along with the p130-Ef24-Sin3a repressor complex or acts independently to repress Sox2 expression is not known. In neural stem cells, p21 is not part of the p130-Ef24-Sin3a repressor complex, which implies that p21 may also act independently in mES cells (Marqués-Torrejón et al., 2013).

## Conclusions and open questions

Pluripotent stem cells exhibit a highly modified cell cycle which allows for rapid proliferation, keeping pace with the requirement for new cells during embryonic development. This rapid proliferation is tightly interlinked to the pluripotent state. As the embryo progresses through development and develops form, the pluripotent cells differentiate to multipotent cells accompanied by lengthening of the cell cycle resulting in reduced proliferation rates. The lengthening of cell cycle is primarily due to the increase in the length of G1 by the activation of RB and Cdkns. Eventually the multipotent progenitors generated during sequential developmental stages differentiate to specific cell types that do not divide i.e., undergo irreversibly exit from the cell cycle. A small sub-population undertakes an alternative program to preserve proliferative capacity, forming committed progenitor cells (adult stem cells) that reversibly exit the cell cycle while retaining their lineage memory or potency, but holding it in reserve until called upon for homeostatic tissue repair and regeneration at later stages. The molecular mechanisms that regulate these transitions seen in the cell cycle during development are still not clearly understood.

Embryonic stem cells are a useful model system to dissect these mechanisms, given their ability to differentiate to most cell types coupled with their limitless ability to self-renew. However, while directed differentiation protocols currently available readily generate fully differentiated cell types such as cardiomyocytes or neurons, the ability to capture cycling committed progenitor states in culture is limited. Therefore, the cell cycle changes that accompany transitions from multipotent to monopotent progenitors are still poorly understood.

By using a combination of population and single-cell techniques, our understanding of the unique regulatory systems controlling the ES cell cycle has improved, but there are still several aspects that require illumination. Key amongst these is the role of the pluripotency factors and lineage determination factors in regulating the cell cycle. While we have highlighted current facets in this review, there are many unexplored aspects which are likely to emerge in the context of reprogramming somatic cells to a pluripotent state.

Further, the therapeutic potential of ES/iPS cells for regenerative medicine makes it imperative to understand the detailed molecular mechanisms at work in regulating the rapid cell cycle. Given that several cell cycle modifications seen in ES cells such as modified cell cycle checkpoints are also seen in cancer cells, it would be important to completely understand these mechanisms before ESC-derived cell types are utilized for cell/tissue replacement in regenerative medicine. Dissecting these pathways may also increase our understanding of tumorigenesis and could lead to potential anti-cancer therapies. At the other end of the disease spectrum, the ability to reactivate non-functioning adult stem cells may ameliorate aspects of aging or degenerative disease. In summary, while a great deal has been learned about both the unique stem cell cycle and pluripotency, the field is poised to achieve an integrated understanding of their developmental context, with wider application of conceptual and technical advances already in play.

## Author contributions

LZ and JD drafted the article and outlined the key concepts to be reviewed. LZ reviewed the literature and prepared all figures and tables. LZ and JD edited and finalized the manuscript.

### Conflict of interest statement

The authors declare that the research was conducted in the absence of any commercial or financial relationships that could be construed as a potential conflict of interest.
